# Effects of Nd_2_O_3_ Nanoparticles on the Structural Characteristics and Dielectric Properties of PVA Polymeric Films

**DOI:** 10.3390/polym15204084

**Published:** 2023-10-14

**Authors:** Khulaif Alshammari, Thamer Alashgai, Alhulw H. Alshammari, Mostufa M. Abdelhamied, Satam Alotibi, Ali Atta

**Affiliations:** 1 Physics Department, College of Science, Jouf University, P.O. Box 2014, Sakaka 72388, Saudi Arabia; 421100043@ju.edu.sa (T.A.); ahalshammari@ju.edu.sa (A.H.A.); aamahmad@ju.edu.sa (A.A.); 2 Charged Particles Lab., Radiation Physics Department, National Center for Radiation Research and Technology (NCRRT), Egyptian Atomic Energy Authority (EAEA), Cairo 13759, Egypt; m_elbana52@yahoo.com; 3 Department of Physics, College of Science and Humanities in Al-Kharj, Prince Sattam bin Abdulaziz University, Al-Kharj 11942, Saudi Arabia; sf.alotibi@psau.edu.sa

**Keywords:** PVA/Nd_2_O_3_, structural investigation, dielectric properties, energy applications

## Abstract

Polyvinyl alcohol (PVA) and Neodymium (III) oxide (Nd_2_O_3_) were combined to synthesized flexible innovative PVA/Nd_2_O_3_ polymer composite samples utilizing a solution casting approach for use in dielectric devices. The XRD, FTIR, and SEM methods are all investigated to characterize the composite films. In a frequency of 50 Hz to 5 MHz, the effects of additive Nd_2_O_3_ on the dielectric behavior of PVA were recorded. The PVA/Nd_2_O_3_ composite films were successfully fabricated, as shown by XRD and infrared spectroscopy. The scanning microscopy pictures showed that the Nd_2_O_3_ was loaded and distributed uniformly throughout the PVA. After the incorporation of Nd_2_O_3_, the composite PVA/Nd_2_O_3_ has a conductivity of 6.82 × 10^−9^ S·cm^−1^, while the PVA has a conductivity of 0.82 × 10^−9^ S·cm^−1^. Another improvement is the decrease in the relaxation time from 14.2 × 10^−5^ s for PVA to 6.35 × 10^−5^ s for PVA/Nd_2_O_3_, and an increase in the dielectric constant of 0.237 for PVA to 0.484 at a frequency of 100 Hz. The results showed that the composite samples have considerable changes as flexible films in different applications, including batteries and electronic circuits.

## 1. Introduction

The use of different polymers, i.e., Polyvinyl chloride (PVC), Polyvinylpyrrolidone (PVP), Polyvinyl alcohol (PVA), etc., has recently increased due to their distinctive properties such as being lightweight and transparent [1]. Their mechanical, optical, or electrical behavior can be influenced by the addition of nanoparticles, resulting in polymer nanocomposites (PNs) [2]. The polymer nanocomposites are launched on the polymer or the polymer matrix which includes the nanofillers within suitable ratios. The weight of the nanofillers is usually far less than the weight of the polymers because some nanofillers are expensive, and they may also have a negative impact on the transparency of polymer films. The efficiency of the polymer nanocomposites’ material in inhibiting electromagnetic interference (EMI) depends on a number of factors, including its dielectric permittivity and electrical conductivity. Dielectric loss, dielectric constant, and their frequency dependencies [3] are all measures of an EMI binding material’s ability to absorb or scatter EM waves. Polymer composites may be a viable alternative to metals and ceramics as a shielding material due to their thermal stability, mechanical performance, and cost [4].

Polymeric materials have become a well-known hosting matrix for rare earth ions because of their attractive advantages, including the ease of fabrication, in addition to their good transparency to the visible and infrared regions [5]. PVA is a polymer that features a carbon chain and hydroxyl group. The PVA polymer has outstanding characteristics such as humidity, water absorption, and being easily produced, which has motivated further studies with this substance [6]. In recent decades, the MOS (metal oxide semiconductor) structure has gained popularity in the field of solid-state electronics. It is simple to examine the dielectric behavior of the insulating oxide layer [7] and the material’s potential compatibility by microelectronic applications [8] using MOS capacitor designs. The ability to modify the properties of nanocomposites has been made possible by incorporating inorganic nanofillers into the polymer. Polymer chains can be altered at their very core thanks to the nanofillers conducting connections within the polymer [9]. Nanoparticle fillers are more efficient than micron-sized fillers; therefore, their integration into polymers results in better polymer characteristics. Variations in the polymer chains’ dielectric properties can be observed as a direct result of the differences in their structural properties [10]. Polymer nanocomposites (PNs) are applied to various promising devices such as nanotechnology and supercapacitors [11]. The doping polymer matrix with suitable nanoparticles will generate PNs with distinctive properties, including mechanical, optical, or electrical properties. Dopants can be rare earth elements such as metal oxide or neodymium oxide (Nd_2_O_3_), which are characterized by their excellent properties. 

The most attention has been paid to rare earth oxides because they have many promising applications. In addition, among the lanthanide series, neodymium oxide (Nd_2_O_3_) is regarded as the most important rare earth oxide. Nd_2_O_3_ is a rare earth element that has been used in sophisticated applications including magnetic devices and protective coatings because of its desirable property [6]. Because of its high dielectric constant, Nd_2_O_3_ is essentially electrically and thermally stable. In addition, its conduction band is well suited for use in microelectronics [12]. These features make Nd_2_O_3_ a superior gate dielectric for semiconductor devices compared to silicon dioxide (SiO_2_). In addition, Nd_2_O_3_ has been extensively applied in optoelectronics because of its narrower gap energy [13]. Several research efforts have focused on studying the alterations in the oxidation properties of rare earth metals through the introduction of various photonics materials, including optical crystals (e.g., Al), glasses, and semiconductors (e.g., Si, SiGe) [14]. Neodymium oxide has shown promising uses in a variety of fields in recent years. This is because neodymium, unlike most rare earth elements, is abundant in the Earth’s crust and rapidly forms its oxides. Crystals of Nd_2_O_3_, a compound with a light grayish blue color, are employed in the production of solid-state lasers. Sunglasses, welding goggles, and other types of glass benefit from Nd_2_O_3_ dichroic characteristics [15].

Moreover, neodymium oxide (Nd_2_O_3_) nanoparticles have garnered a lot of attention among the rare earth elements due to their superior dielectric characteristics. In comparison to excited electrons, the UV- excitation produces more effective light radiation [16]. In recent times, there has been a significant focus on Nd_2_O_3_ nanoparticles as a dielectric filler due to their outstanding properties that have potential applications in various fields such as thermo-luminescence, protective coatings, and dielectric devices [17]. The nanoparticles of Nd_2_O_3_ have several potential applications because of their size impact. Therefore, Nd_2_O_3_ nanoparticle synthesis has received considerable interest in recent years [18]. Keikhaei et al. have investigated the incorporation of Nd_2_O_3_ on the properties of the PVA polymer [19]. Various characterizations, namely structural, electrical, and optical, were conducted on the PVA/PVP blend doped with Nd_2_O_3_ [6]. AlAbdulaal et al. [20] studied the optical behavior for PVA doped by various quantities of Nd_2_O_3_, which were processed by the casting method. The novelty of this research is to improve the electrical characteristics of the PVA polymer doped by Nd_2_O_3_ nanoparticles, which have hardly been studied by other researchers. Therefore, the present research modified the dielectric characteristics of PVA for applications in energy devices. The XRD, SEM, and FTIR methods were used to investigate the PVA/Nd_2_O_3_ characteristics. In a frequency spanning from 100 Hz to 5 MHz, the permittivity, impedance, and dielectric efficiency of PVA/Nd_2_O_3_ films were measured.

## 2. Materials and Methods 

The Nd_2_O_3_ powder (purity of 99.95% and average size of 25–40 nm) was purchased from Nanografi Nano Technology Company (Darmstadt, Germany). The PVA powder (molecular weight: 29,000–69,000 g/mol, and 87.90% hydrolyzed) was given by Sigma-Aldrich Company, Darmstadt, Germany. The solution casting method [21,22] was elected for making polymer films because it involves a simple preparation, is inexpensive, allows for the precise management of composite composition, and produces highly uniform nanocomposite films. Firstly, 1.0 g of PVA was dissolved in 85 mL of deionized water with magnetic stirring for 1.5 h. After stirring the PVA polymer for 1.5 h, different amounts of Nd_2_O_3_ (0.05, 0.10, 0.15, and 0.20 wt%) were added and mixed in PVA. The PVA/Nd_2_O_3_ solution was then poured into a glass Petri dish and allowed to air dry at room temperature. The sheets were then removed from the glass plates with an average thickness of 170 μm.

The chemical changes were studied with FTIR (Shimadzu FTIR, Tracer 100, Kyoto, Japan) in the wavenumber of 410–3900 cm^−1^, while XRD (Shimadzu XRD 7000, Kyoto, Japan) was used to examine the structural characteristics at 2θ range (10–80)°. In order to determine the morphology and geometric shape of the films, FESEM (Thermo Fisher Scientific, Waltham, MA, USA) was used. Typically, a gold film is sputtered onto the surface of the film before the sample is placed under an electron microscope. The LCR meter (RS-232C interface, Hioki, Japan) is used to record the dielectric parameters at room temperature at a frequency of 50 Hz to 5 MHz.

## 3. Results and Discussion

**Figure 1:**
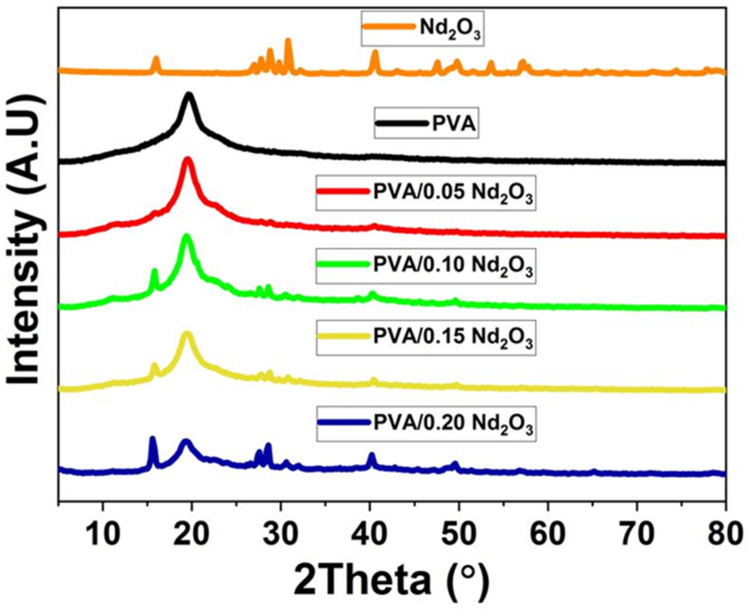
XRD diffraction for the polymer PVA, Nd_2_O_3_, and the composite PVA/Nd_2_O_3_.

The XRD was utilized for studying the structure of the PVA film and PVA/Nd_2_O_3_ nanocomposite, as shown in Figure 1. The pattern of the PVA displayed a peak located at 2θ=19.6°, which refers to the (101) plane. A significant peak is observed at approximately 2θ=40°, indicating that the structure of (PVA) exhibits semi-crystalline characteristics [23]. The XRD peaks observed of Nd_2_O_3_ were found to be consistent with the standard reference card for Nd_2_O_3_ with JCPDS Card No: 74–1147. The distinct diffraction peak observed in the XRD pattern of Nd_2_O_3_ indicates the presence of an orthorhombic lattice structure. Furthermore, it has been observed that the peak at 19.4° exhibits a decrease by the incorporation of Nd_2_O_3_. The inclusion of Nd_2_O_3_ causes modifications to the amorphous structure of the PVA, resulting in a change in crystallinity [24]. In addition, the number of planes of the peaks’ angles is modified as Nd_2_O_3_ concentration increases, indicating that the PVA and Nd_2_O_3_ nanoparticles interacted [25]. The addition of Nd_2_O_3_ to PVA results in higher crystallinity in comparison to pure PVA. X-ray diffraction (XRD) analysis revealed the formation of a complex between the Nd_2_O_3_ additives and the polyvinyl alcohol (PVA) polymer chain.

The crystallite size of Nd_2_O_3_ is determined by using the Scherer formula [26].

(1) $$D=\frac{0.9\lambda }{\beta cos\theta }$$

The crystallite size is *D*, *β* is the full width, and λ is the wavelength. With increasing concentrations of PVA, the crystallite size of Nd_2_O_3_ grows from 13.5 nm for PVA/0.1Nd_2_O_3_ to 15 nm and 18.2 nm, respectively, for PVA/0.15Nd_2_O_3_ and PVA/0.2Nd_2_O_3_, demonstrating that Nd_2_O_3_ is formed at the nanoscale. Because of its low concentration (0.05 wt%), PVA/0.05Nd_2_O_3_ does not exhibit any peaks characteristic of Nd_2_O_3_ in XRD spectra. In turn, this makes it easier for Nd_2_O_3_ to be distributed uniformly throughout the PVA. 

**Figure 2:**
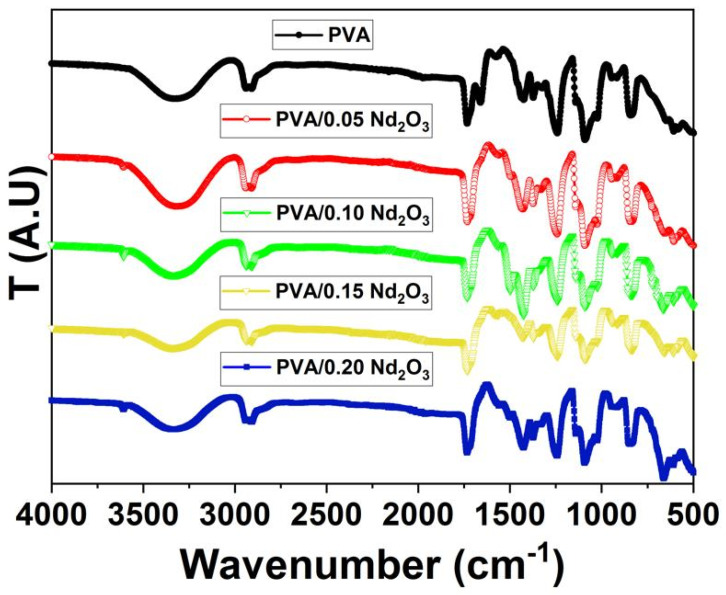
FTIR spectrum for polymer nanocomposite PVA/Nd_2_O_3_ and different concentrations of Nd_2_O_3_.

An additional investigation through the utilization of FTIR analysis was conducted to prove the changes occurred in the chemical characteristics of the composite after the incorporation of Nd_2_O_3_. Furthermore, FT-IR was utilized to analyze the functional groups present in the composite films of PVA doped with the Nd_2_O_3_ nanocomposite. The FT-IR measurements were obtained from 4000 to 500 cm^−1^ in wavenumber, as shown in Figure 2. The FTIR of PVA showed a band observed at a wavenumber of 3285 cm^−1^, with the stretching O–H resulting from the hydrogen bonds between and inside molecules, as reported in previous studies [27]. Moreover, for the C–H band, asymmetric stretching vibrations are found at a wavenumber of 2920 cm^−1^. The bands of 1720 cm^−1^ and 1080 cm^−1^ correspond to carbonyl stretching (C=O) and C-O bond stretching from the acetate groups. C-H bending appeared in the infrared (IR) region at 1430 cm^−1^. The wavenumber at 840 cm^−1^ provided evidence of the specific formation of CH_2_ bending [28]. The observed reduction in the maximum intensity of PVA due to the addition of Nd_2_O_3_ indicates the presence of intermolecular bands, which can be attributed to the hydroxyl group present in PVA. The Nd_2_O_3_ nanoparticle incorporation changed the electron-hole recombination rate because of the defects created into the PVA polymeric structure, leading to a corresponding change in the peak positions. At the same time, the ability of Nd_2_O_3_ to dampen the brightness of PVA/Nd_2_O_3_ indicates that there are too many electrons present, leading to enhanced performance in terms of the materials’ electrical properties.

**Figure 3:**
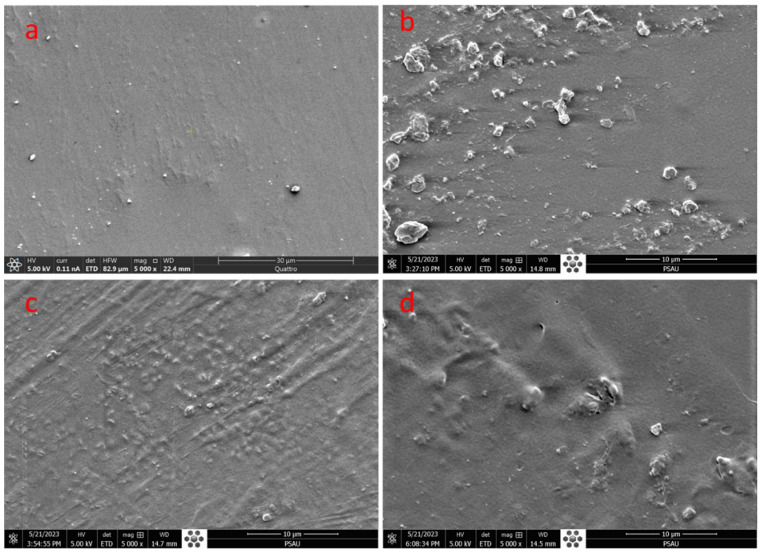
SEM of (**a**) PVA, (**b**) PVA/0.05 Nd_2_O_3_, (**c**) PVA/0.10Nd_2_O_3_, and (**d**) PVA/0.20Nd_2_O_3_.

The morphology of PVA/Nd_2_O_3_ is shown in Figure 3a–d. A micrograph of PVA film, as shown in Figure 3a, has a uniform and smooth surface. Figure 3b–d depict PVA/Nd_2_O_3_ films with 0.05Nd_2_O_3_, 0.10Nd_2_O_3_, and 0.20Nd_2_O_3_. The SEM pictures of the composites reveal no discernible agglomeration, cracks, or breaks, suggesting that the cross-linker is well dispersed in the PVA chains by increasing Nd_2_O_3_ [28]. The increase in Nd_2_O_3_ content (from 5% to 20%) causes the dispersion to become highly apparent, as clearly shown in Figure 3b–d. The presence of the cross-linkers created in the composite is attributed to the formation of strong hydrogen bonds between PVA and Nd_2_O_3_ or the increase in viscosity during the composite preparation. This phenomenon has been documented in previous investigations [5,20,28]. It is possible that the observed morphological alterations are due to Nd_2_O_3_ and the preparation method conditions. The concentration of Nd_2_O_3_ in PVA also affects its dispersion and the interfacial interaction of PVA chains and Nd_2_O_3_ [5]. 

The dielectric real $\varepsilon^{\prime }$ and imaginary ε″ value, which are related to complex dielectric permittivity (ε*), are given by [29]:

(2) $$\varepsilon^{*}=\varepsilon^{\prime }-i\;\varepsilon \prime \prime$$

**Figure 4:**
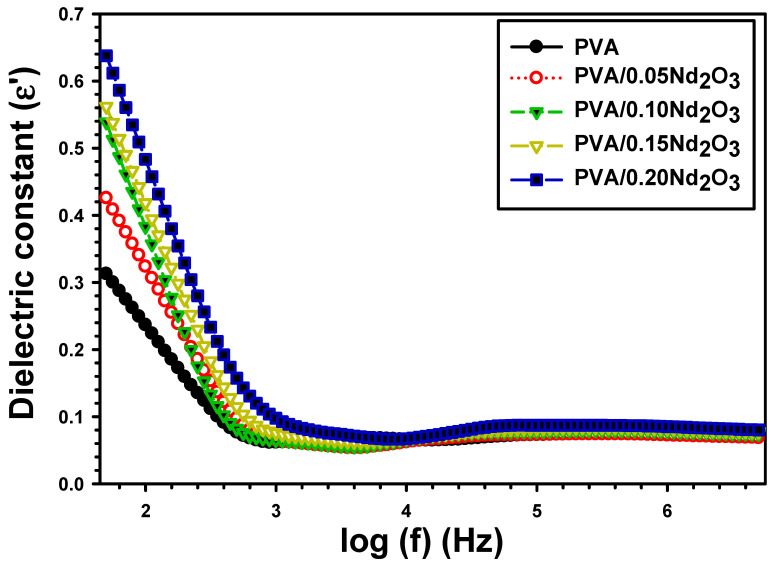
The dielectric constant $\left(\varepsilon^{\prime }\right)$ with frequency for PVA and different concentrations of Nd_2_O_3_ nanocomposite.

The real $\varepsilon^{\prime }$ dielectric constant $\varepsilon^{\prime }$ is given by [30]:

(3) $$\varepsilon^{\prime }=\frac{c\;.\;\;d}{\varepsilon_{o}\;.\;A}$$

c is the capacitance, the film thickness is t, and A is the area. Figure 4 displays the $\;\varepsilon^{\prime }$ with frequency for the PVA, PVA/0.05Nd_2_O_3_, PVA/0.10Nd_2_O_3_, PVA/0.15Nd_2_O_3_, and PVA/0.20Nd_2_O_3_ films. At a lower frequency, the frequency-dependent attenuation of the $\varepsilon^{\prime }$ is observed for all samples. This occurs because the dipoles have enough time to encounter polarization between themselves at smaller frequencies. Then, the $\;\varepsilon^{\prime }$ remains nearly constant at higher frequencies, which may be because the dipoles do not have more time to align with the orientation of the external fields [31]. In addition, the high frequency values of the applied external fields are too rapid for the interface states to rearrange.

Incorporating Nd_2_O_3_ increases the $\;\varepsilon^{\prime }$ from 0.236 for pure PVA at 100 Hz to 0.323, 0.383, 0.418, and 0.483 for PVA/0.05 Nd_2_O_3_, PVA/0.10 Nd_2_O_3_, PVA/0.15 Nd_2_O_3_, and PVA/0.20 Nd_2_O_3_ (Table 1). The rapid growth due to Nd_2_O_3_ also increases dipole density and hence, enhances polarizability at interfaces. Thus, the increased polarization and dielectric characteristics leads to an increased number of dipoles caused by the flaws in the PVA polymer matrix. Atta [32] also showed this phenomenon when CuNPs were deposited on PTFE and PET polymeric films. He showed that the $\;\varepsilon^{\prime }\;$ increased from 0.63 for pure PET to 1.18 after 25 min of deposition Cu on PET, and from 1.95 for pure PTFE to 2.25 for Cu on PTFE. The improvement in the dielectric constant supports the use of these samples in energy storage applications with a high ability to store charge.

The imaginary dielectric loss $\varepsilon^{\prime \prime }$ is given by [33]:

(4) $$\varepsilon^{\prime \prime }=\varepsilon^{\prime \;}\tan\delta$$

**Figure 5:**
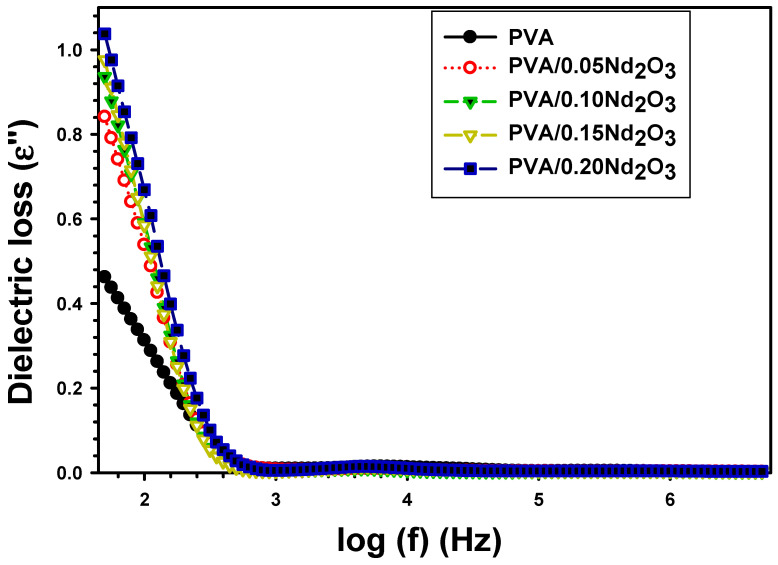
The dielectric loss (ε′′) with frequency for PVA and different concentrations of Nd_2_O_3_ nanocomposite.

Figure 5 illustrates the frequency-dependent dielectric loss $\varepsilon^{\prime \prime }$ for PVA, PVA/0.5Nd_2_O_3_, PVA/0.10Nd_2_O_3_, PVA/15Nd_2_O_3_, and PVA/0.20Nd_2_O_3_. It has been shown that the enhancement of the frequency significantly reduces the dielectric loss at a lower frequency [34,35]. This shift in $\varepsilon^{\prime \prime }$ is also associated with the low-frequency active characteristic trait of dipolar relaxation. Charge carriers is another explanation for the varying of $\varepsilon^{\prime \prime }$ with frequency. Furthermore, lowering the frequency triggers a charge transfer across the interface. It has been established that PVA/0.05Nd_2_O_3_ and PVA/0.20Nd_2_O_3_ both increase the $\varepsilon^{\prime \prime }$ value from 0.313 for PVA to 0.539 and 0.669, respectively. The improved PVA chain and Nd_2_O_3_ links are responsible for the increased $\varepsilon^{\prime \prime }$ with the addition of Nd_2_O_3_ resulting in a more robust coupling across the grain boundary [35]. The mini-capacitor networks formed in PVA/Nd_2_O_3_ films are also responsible for the enhancement in the dielectric value.

The electrical modulus (M*) is estimated by [35]:

(5) $$M^{*}=\frac{1}{\varepsilon^{*}}=M\prime +i\;M\prime \prime$$

The M′ and M″ are, respectively, the real and imaginary electric modulus, and are recorded by [36]:

(6) $$M\prime =\frac{\varepsilon^{\prime }}{\varepsilon^{\prime 2}+\varepsilon^{\prime \prime 2}}$$

(7) $$M\prime \prime =\varepsilon \prime \prime /\left(\varepsilon^{\prime 2}+\varepsilon^{\prime \prime 2}\right)$$

**Figure 6:**
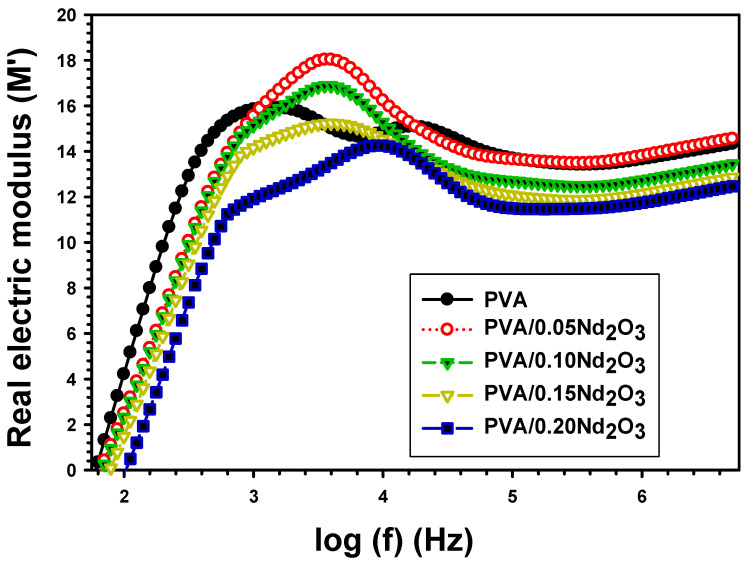
The real electric modulus (M′) with frequency for PVA and PVA/%Nd_2_O_3_ films.

**Figure 7:**
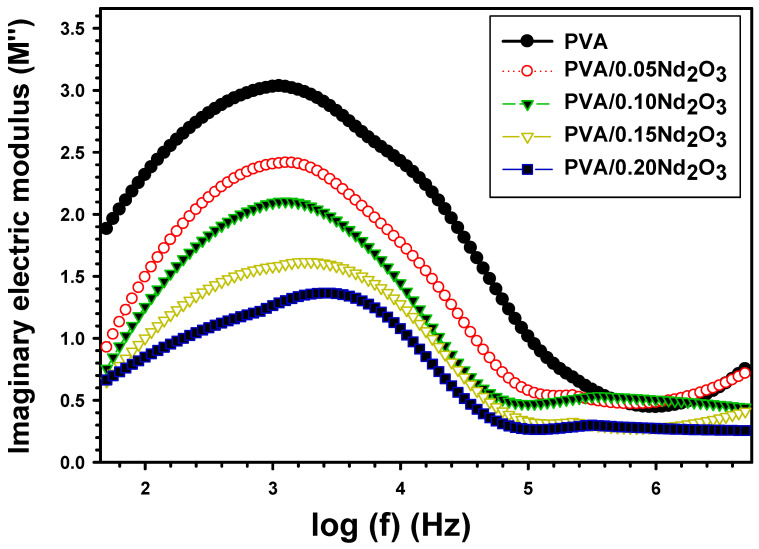
The imaginary electric modulus (M′′) versus frequency for PVA and PVA/%Nd_2_O_3_ films.

Figure 6 displays the M′ versus frequency with different concentrations of Nd_2_O_3_. M′ increases exponentially with frequency, and at a higher frequency, it becomes straight and constant. The polymer electrical dipoles have a considerable self-orientation propensity at low frequencies, while the orientation produced by an external electric field is minimal at high frequencies [37]. The M′ is also affected by the electrode polarization [38]. As can be shown in Figure 7, the incorporation of Nd_2_O_3_ causes a decrease in M′. The value of M′ of PVA is 4.21 at 100 Hz, and it dropped to 2.488, 2.318, 1.469, and 0.487 when doped with varying concentrations of Nd_2_O_3_. The permittivity for the electric modulus reduces due to the dipolar contribution. The dispersion of charges in the oxide may also play a role in this attenuation.

Figure 7 displays the imaginary modulus M″ for PVA, PVA/0.05Nd_2_O_3_, PVA/0.10Nd_2_O_3_, PVA/0.15Nd_2_O_3_, and PVA/0.20Nd_2_O_3_. A peak in M″ refers to the presence of a relaxation process (as depicted in the picture). Higher permittivity is the result of an oriented M″ value at a low frequency, which indicates that the ions are transferred by hopping from one location to another. With an increase in frequency, the orientation of more dipolar groups becomes more challenging. The M″ of PVA is 2.318 at 100 Hz, but it drops to 1.493, 1.255, 1.01, and 0.848 when doped with 0.05Nd_2_O_3_, 0.10Nd_2_O_3_, 0.15Nd_2_O_3_, and 0.20Nd_2_O_3_. This is because the charged interface region is formed of PVA and Nd_2_O_3_. The time of relaxation ($\tau_{r}$) is estimated by the following relation [39]:

(8) $$\tau_{r}=\frac{1}{\omega_{r}}$$

where $\tau_{r}$ is the angular frequency that corresponds to the crest of the M″ peak. The relaxation time, measured in seconds, drops from 14.2 × 10^−5^ s for pure PVA film to 12.67 × 10^−5^ s for PVA/0.05Nd_2_O_3_, to 11.29 × 10^−5^ s for PVA/0.10Nd_2_O_3_, and finally to 8.97 × 10^−5^ s and 6.35 × 10^−5^ s for PVA/0.15Nd_2_O_3_ and PVA/0.20Nd_2_O_3_. This trend arises because the relaxation duration varies between the acceptor- and donor-of-interface states. Abdelhamied et al. [40] found that the relaxation time for PVA/PANI/Ag is changed by frequency. They discovered that the PVA film had a relaxation time of 3.3 × 10^−5^ s, but this reduced to 3.25 × 10^−5^ s and 1.9 × 10^−5^ s, respectively, for PVA/PANI and PVA/PANI/Ag. The Nd_2_O_3_ shortens the time needed for dipole orientation. The hopping process directly enhanced dipole mobility, which in turn decreased M″ [41]. 

The complex impedance of PVA and PVA/Nd_2_O_3_ is given by [42]:

(9) $$Z^{*}=Z^{\prime }+iZ^{\prime \prime }$$

**Figure 8:**
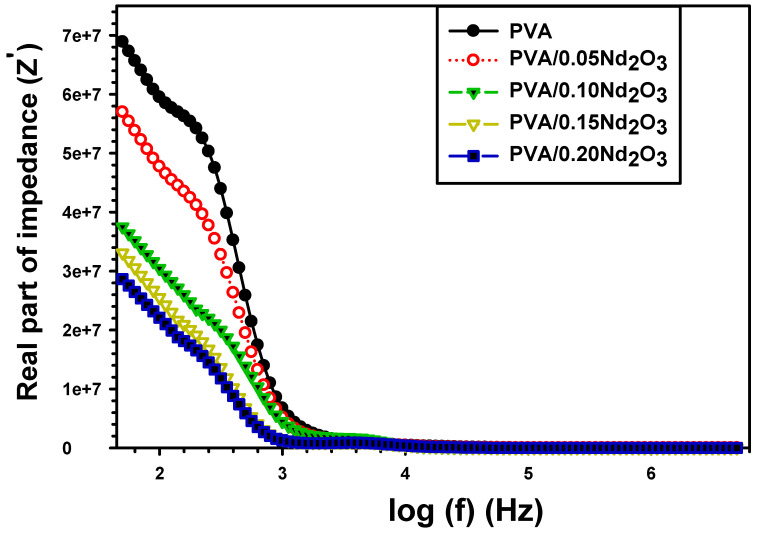
The real impedance $\left(Z^{\prime }\right)$ with frequency for PVA and PVA/%Nd_2_O_3_ films.

Z* stands for the complex impedance, $Z^{\prime }$ for the real impedance, and Z″ for the imaginary impedance. Figure 8 shows that the real $Z^{\prime }$ impedance of pure PVA and PVA/0.20Nd_2_O_3_ films varies as a function of frequency. In the lower frequency, the $Z^{\prime }$ of all samples decreases significantly with frequency and is often fixed in the high frequency range. This behavior is explained by the fact that an enhancement in the number of free charges causes the conductivity to rise and the impedance to remain stable [43]. The addition of Nd_2_O_3_ has the opposite effect, progressively lowering the Z′ value. The inclusion of Nd_2_O_3_ enhanced conductivity and directly decreased the films’ impedance, leading to a decrease in Z′. Notably, the occurrence of peaks in PVA/Nd_2_O_3_ films is confirmed to be an improvement in the composite films, making them more suitable for devices.

**Figure 9:**
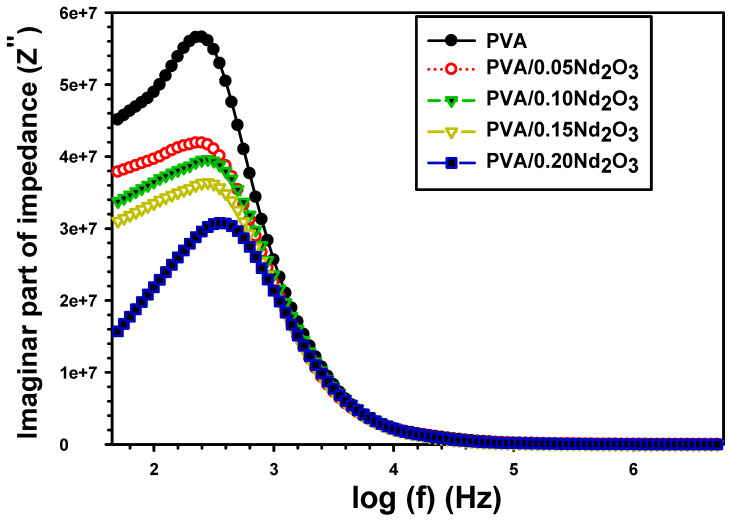
The imaginary impedance $\left(Z^{\prime \prime }\right)$ with frequency for PVA and PVA/%Nd_2_O_3_ films.

Figure 9 displays the relationship between the imaginary impedance component Z″ and frequency for films of pure PVA, PVA/0.05Nd_2_O_3_, PVA/0.10Nd_2_O_3_, PVA/0.15Nd_2_O_3_, and PVA/0.20Nd_2_O_3_. The Z″ drops by frequency. Incorporating more Nd_2_O_3_ into PVA decreases the Z″ values, resulting in a faster charge transfer compared to pure PVA [44]. 

The energy density (U) as a function with $\;\varepsilon^{\prime }$ is given by [45]:

(10) $$U=\frac{1}{2}\;\varepsilon^{\prime }\varepsilon_{O}\;E^{2}$$

**Figure 10:**
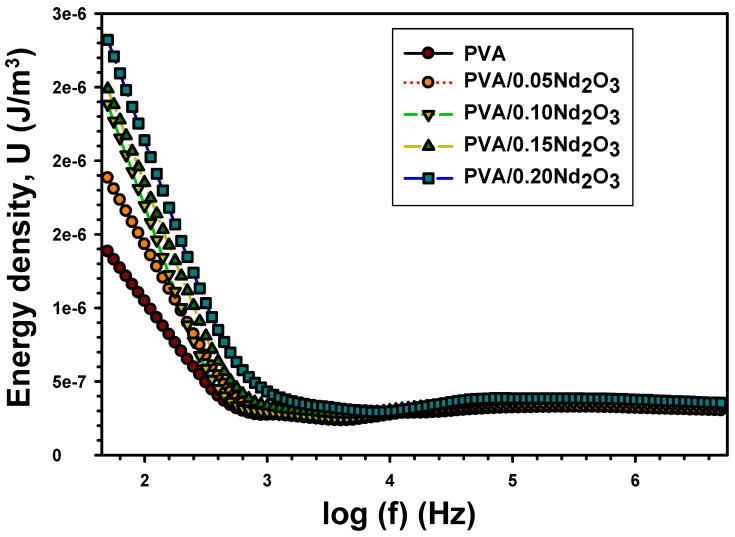
The energy density (U ) with frequency for PVA and PVA/%Nd_2_O_3_ films.

*E* is the electric field, which is assumed to be 1 V/mm, and $\varepsilon_{O}$ is the electric permittivity, which is equal to 8.85×10^−11^ NVm. The energy density at various frequencies for pure PVA, PVA/0.05Nd_2_O_3_, PVA/0.10Nd_2_O_3_, PVA/0.15Nd_2_O_3_, and PVA/0.20Nd_2_O_3_ films are displayed in Figure 10. Interface states that vary in strength with frequency are one possible explanation for this pattern. Since the contents of Nd_2_O_3_ increase, PVA had a higher power density. Table 1 shows that the addition of varying amounts of neodymium oxide into PVA results in an enhancement of its energy density. Particularly, the energy density values increase from 1.05 × 10^−6^ J/m^3^ (the value for pure PVA) to 1.43 × 10^−6^ J/m^3^, 1.70 × 10^−6^ J/m^3^, 1.85 × 10^−6^ J/m^3^, and 2.14 × 10^6^ J/m^3^, respectively. One potential explanation for this phenomenon could be the presence of border traps in close proximity to the interface between Nd_2_O_3_ and PVA. These findings provide further evidence of the effective incorporation of Nd_2_O_3_ within PVA.

The conductivity ($\sigma_{ac})$ as a function of the dielectric constant is given by [46]:

(11) $$\sigma_{ac}=\varepsilon_{o}\varepsilon^{\prime \prime }\omega$$

**Figure 11:**
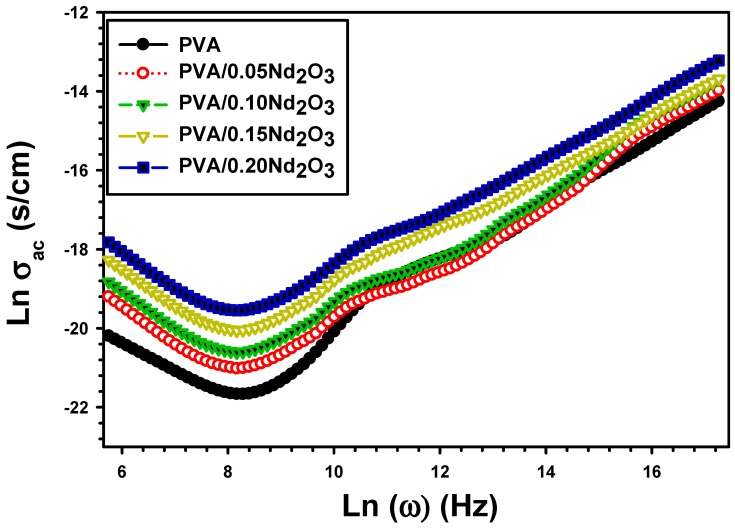
The conductivity ($\sigma_{ac})$ with frequency for PVA and PVA/%Nd_2_O_3_ films.

ω is the angular frequency and $\varepsilon_{o}$ is permittivity [47]. Films made of pure PVA, PVA/0.05Nd_2_O_3_, PVA/0.10Nd_2_O_3_, PVA/0.15Nd_2_O_3_, and PVA/0.20Nd_2_O_3_ show different changes in electrical conductivity as the frequency increases from 100 Hz to 5 MHz (Figure 11). In addition, the activation of trapped charges results in a shift in $\sigma_{ac}$ conductivity [48]. According to Table 1, the electrical conductivity of PVA at 100 Hz ranges from 0.82 × 10^−9^ S/cm for pure PVA to 1.67 × 10^−9^ S/cm for PVA/0.05Nd_2_O_3_ and 6.82 × 10^−9^ for PVA/0.20Nd_2_O_3_.

Moreover, the addition of Nd_2_O_3_ causes the greatest improvement in electrical conductivity because chain scissoring causes an increase in the speed of transported ions [49]. The conductivity of the PVA/MWCNT composite is investigated by Atta et al. [50]. The $\sigma_{ac}$ of PVA is 0.89 × 10^−4^ S/cm at 1 MHz, while the $\sigma_{ac}$ of PVA/MWCNT increased to 2.6 × 10^−6^ S/cm. This is because the MWCNT-induced increase in the carrier’s charges led to the coalescence of grain boundaries, which improved conductivity.

The potential barrier height *W_m_* is estimated by [51]:

(12) $$W_{m}=\frac{-4k_{B}T}{m}$$

**Figure 12:**
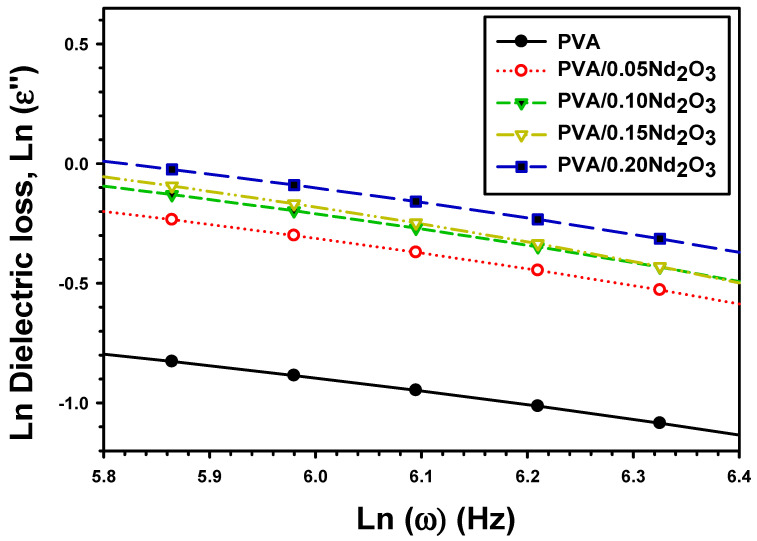
Variation of Ln ($\sigma_{ac}$) versus Ln (ω) for PVA and PVA/%Nd_2_O_3_ films.

*k_B_* is the Boltzmann constant and *m* is given by the slope of *lnε_2_* and *lnω*, as investigated in Figure 12 by [52]:

(13) $$\varepsilon^{\prime \prime }=A\omega^{m}$$

where $\varepsilon^{\prime \prime }$ is the dielectric loss and ω is the angular frequency. As the percentage of Nd_2_O_3_ increased in the formula, the predicted potential barrier $W_{m}$ is modified. The $W_{m}$ energy of PVA with various concentrations of neodymium oxide varies from 0.264 eV to 0.248 eV and 0.255 eV for pure PVA, and to 0.284 eV and 0.252 eV for PVA/0.05Nd_2_O_3_, PVA/0.10Nd_2_O_3_, PVA/0.15Nd_2_O_3_, and PVA/0.20Nd_2_O_3_. The XRD measurements show that the inclusion of Nd_2_O_3_ alters the crystal structure and increases the defect density, leading to a lower potential barrier. Therefore, this evidence of a lower energy barrier for charge carrier hopping demonstrates that increasing the Nd_2_O_3_ content in PVA improves its $\sigma_{ac}$ electrical conductivity.

## 4. Conclusions

The casting reduction method was used to successfully create PVA/Nd_2_O_3_ composite films, which were then characterized using a variety of methods. The XRD and FTIR methods verify that PVA/Nd_2_O_3_ was successfully synthesized. The inclusion of Nd_2_O_3_ causes modifications to the amorphous structure of the PVA, resulting in a change in PVA crystallinity. Moreover, SEM analysis shows that Nd_2_O_3_ is well incorporated into the PVA. It is observed that the morphology is altered due to Nd_2_O_3_. The concentration of Nd_2_O_3_ in PVA also affects its dispersion and the interfacial interaction of PVA chains and Nd_2_O_3_. The inclusion of Nd_2_O_3_ has also been observed to improve electrical conductivity and dielectric characteristics. The addition of Nd_2_O_3_ causes the greatest improvement in electrical conductivity because chain scissoring causes an increase in the speed of transported ions. In addition, there was a correlation between the amount of Nd_2_O_3_ and the changes in the electric modulus and complex impedance. Dielectric dispersion qualities were enhanced as Nd_2_O_3_ concentrations were raised from 0.05 wt% to 0.20 wt%, thanks to the activity of charge carriers. The electric modulus increases exponentially in a low frequency region, and at a higher frequency it becomes nearly constant. This is because the polymer electrical dipoles have a considerable self-orientation propensity at low frequencies, while the orientation produced by an external electric field is minimal at high frequencies. Based on the electrical results, the produced results have paved the way for using flexible PVA/Nd_2_O_3_ samples in a wide variety of possible applications, including batteries, super-capacitors, and microelectronic devices.

## Figures and Tables

**Table 1:** Values of $\varepsilon^{\prime }$, ε″, $\sigma_{\mathrm{ac}}$, $M^{\prime }$, M″, and U of PVA and PVA/Nd_2_O_3_.

	$\varepsilon^{\prime }$	$\varepsilon^{\prime \prime }$	$M^{\prime }$	$M^{\prime \prime }$	$\sigma_{ac}$ (S/cm)	*U (*J/m^3^*)*
PVA	0.237	0.312	4.21	2.31	0.82×10^−9^	1.05×10^−6^
PVA/0.05Nd_2_O_3_	0.324	0.539	2.48	1.49	1.67×10^−9^	1.43×10^−6^
PVA/0.10Nd_2_O_3_	0.383	0.592	2.31	1.25	2.01×10^−9^	1.70×10^−6^
PVA/0.15Nd_2_O_3_	0.428	0.585	1.46	1.01	4.32×10^−9^	1.8510^−6^
PVA/0.20Nd_2_O_3_	0.483	0.669	0.488	0.84	6.82×10^−9^	2.14×10^−6^

## Data Availability

Data will be made available upon reasonable request.
